# A Practical Treatise on Military Surgery

**Published:** 1861-07

**Authors:** 


### Book Notices.

A PRACTICAL TREATISE ON MILITARY SURGERY. By FRANK HASTINGS HAMILTON, M. D., Author of a "Treatise on Fractures and Dislocations," Surgeon-in-Chief to the Long Island College Hospital, Surgeon to the Bellevue Hospital, New York; Professor of Military Surgery and of Diseases and Accidents incident to Bones, in the Bellevue Hospital. New York: 1861.

We have already noticed in our pages the preparation of this volume, by the distinguished author of "Fractures and Dislocations"—the most complete work of its kind in the language—and though the *Military Surgery* has been preceded by kindred works, we have lost nothing by the delay, since it has given us a volume worthy of its author.

Accurately speaking, however, neither this, nor Gross's, nor Tripler and Blackman's, is a *work on Military Surgery*, but they are all *Hand-Books for the Military Surgeon;* suggestive and practical, rather than exhaustive and disquisitionary, as their titles—with the exception of the last named—would lead one to believe. The volumes are what are needed in the emergency, but their titles are certainly misnomers. And yet Prof. HAMILTON has, we think, gone a step in advance of his co-laborers, and so far as might be, consistently with the urgency of the case and limited space and time, has produced a volume, valuable not only for its practical hints as to the conduct of camp and field hospitals, examination of recruits, mode of making requisitions and the numerous other technical details,—the *opus operatum* of the army surgeon,—but also for its sound rules and counsel in strictly surgical and medical practice. We can give no clearer idea of the particulars in which we think this *Treatise* worthy, than by quoting the author's statement of the deficiencies it is intended to supply—merely premising that the intention is well fulfilled:—

General treatises upon surgery and surgical teachers, assume that both the patient and his medical attendant are placed always under the most favorable circumstances: that ample time is allowed for a careful diagnosis; and, in view of an operation, that the patient is brought up to the best possible condition of preparation: that he is at least comfortably lodged, suitably nourished, and that his surgeon has at his command all the instruments and appliances which can render the execution of the operation more easy, and its success more certain. No man who has had much experience in teaching, and in examining medical students, can have failed to notice the danger of suggesting inferior alternatives for exceptional cases, which, through inattention or carelessness, are often substituted in the minds of the pupil for the general law; and it is with much propriety, therefore, that these omissions are generally made.

It is the special province of military and naval surgery to supply these deficiencies; instructing the pupil how, by a multitude of extemporaneous expedients, he may succor the wounded and relieve the sick when the usual resources fail or are not at hand; how he may make the products of every country contribute to his necessities, and a single cruse of oil minister miraculously to a thousand.

The work embraces a consideration of the Examination of Recruits, the Hygiene of Troops, relating to Diet, Dress, Exercise, etc.; Accommodation of Troops in Tents, Barracks, Huts, etc.; the Construction and Location of Hospitals; Preparations for the Field; Flying Ambulances, Litters, etc.; also, Gunshot Wounds, Amputations, Hospital Gangrene, Scurvy, etc.; and an Appendix containing the United States Army Medical Regulations, with many other matters pertaining to Military Surgery.

The chapter on *Dysentery* is contributed by Prof. Austin Flint, M. D., and that on *Scurvy* by Prof. Benj. W. McCready,

M. D.; while the volume is replete with the experience and dicta of Drs. Satterlee, Wood, Coolidge, Tripler, Mills, Pitcher, Wright and others of the U. S. Army; and Drs. Parsons, Bache, Lockwood, Turner, Williams and others of the Navy. It is fully illustrated and well printed.

Bailliere Brothers, 440 Broadway, N. Y. W. B. Keen, 148 Lake Street, Chicago.

